# Effects of Exercise Intervention on Type 2 Diabetes Patients With Abdominal Obesity and Low Thigh Circumference (EXTEND): Study Protocol for a Randomized Controlled Trial

**DOI:** 10.3389/fendo.2022.937264

**Published:** 2022-07-12

**Authors:** Dan Liu, Ying Zhang, Liang Wu, Jingyi Guo, Xiangtian Yu, Huasheng Yao, Rui Han, Tianshu Ma, Yuchan Zheng, Qiongmei Gao, Qichen Fang, Yan Zhao, Yanan Zhao, Biao Sun, Weiping Jia, Huating Li

**Affiliations:** ^1^ Department of Endocrinology and Metabolism, Shanghai Jiao Tong University Affiliated Sixth People’s Hospital, Shanghai Diabetes Institute, Shanghai Clinical Center for Diabetes, Shanghai Key Laboratory of Diabetes Mellitus, Shanghai, China; ^2^ Department of Medicine, Shanghai Jiao Tong University School of Medicine, Shanghai, China; ^3^ Clinical Research Center, Shanghai Jiao Tong University Affiliated Sixth People’s Hospital, Shanghai, China; ^4^ School of Sports Science and Physical Education, Nanjing Normal University, Nanjing, China; ^5^ Department of Kinesiology, Nanjing Sport Institute, Nanjing, China; ^6^ Department of Sports and Health Science, Nanjing Sport Institute, Nanjing, China

**Keywords:** type 2 diabetes mellitus, exercise, abdominal obesity, thigh circumference, brain health, gut microbiota

## Abstract

**Introduction:**

Type 2 diabetes patients have abdominal obesity and low thigh circumference. Previous studies have mainly focused on the role of exercise in reducing body weight and fat mass, improving glucose and lipid metabolism, with a lack of evaluation on the loss of muscle mass, diabetes complications, energy metabolism, and brain health. Moreover, whether the potential physiological benefit of exercise for diabetes mellitus is related to the modulation of the microbiota-gut-brain axis remains unclear. Multi-omics approaches and multidimensional evaluations may help systematically and comprehensively correlate physical exercise and the metabolic benefits.

**Methods and Analysis:**

This study is a randomized controlled clinical trial. A total of 100 sedentary patients with type 2 diabetes will be allocated to either an exercise or a control group in a 1:1 ratio. Participants in the exercise group will receive a 16-week combined aerobic and resistance exercise training, while those in the control group will maintain their sedentary lifestyle unchanged. Additionally, all participants will receive a diet administration to control the confounding effects of diet. The primary outcome will be the change in body fat mass measured using bioelectrical impedance analysis. The secondary outcomes will include body fat mass change rate (%), and changes in anthropometric indicators (body weight, waist, hip, and thigh circumference), clinical biochemical indicators (glycated hemoglobin, blood glucose, insulin sensitivity, blood lipid, liver enzyme, and renal function), brain health (appetite, mood, and cognitive function), immunologic function, metagenomics, metabolomics, energy expenditure, cardiopulmonary fitness, exercise-related indicators, fatty liver, cytokines (fibroblast growth factor 21, fibroblast growth factor 19, adiponectin, fatty acid-binding protein 4, and lipocalin 2), vascular endothelial function, autonomic nervous function, and glucose fluctuation.

**Discussion:**

This study will evaluate the effect of a 16-week combined aerobic and resistance exercise regimen on patients with diabetes. The results will provide a comprehensive evaluation of the physiological effects of exercise, and reveal the role of the microbiota-gut-brain axis in exercise-induced metabolic benefits to diabetes.

**Clinical Trial Registration:**

http://www.chictr.org.cn/searchproj.aspx, identifier ChiCTR2100046148.

## Introduction

Diabetes mellitus is a global health challenge, which is estimated to affect 783.2 million people by 2045 (1). Abdominal obesity is a common somatotype in patients with type 2 diabetes, while some studies have reported that type 2 diabetes patients also have a low thigh circumference (2, 3), which becomes more obvious with age (4). Low femoral subcutaneous fat is associated with unfavorable glucose and lipid levels (5), and thigh muscle mass area is inversely associated with future insulin resistance (6). Sarcopenia is an age-related process that occurs commonly in the elderly population, with a progressive skeletal muscle disorder involving the accelerated loss of muscle mass and function (7). Currently, sarcopenia in type 2 diabetes, a condition known as secondary sarcopenia, has gained increased attention (8). Exercise is considered as one of the effective therapeutic methods for type 2 diabetes and sarcopenia. Previous studies have mostly focused on the effect of exercise on obesity and visceral fat accumulation in type 2 diabetes, while its action on low thigh circumference has rarely been explored.

In the recent decades, the relationship between diabetes and brain health has gradually attracted attention (9–11). Diabetes has been identified as a key risk factor for mild cognitive impairment and dementia (12). Appetite is also altered in patients with obesity and type 2 diabetes. The brain domains of obese people are more sensitive to hunger and have increased activation of reward regions for visual food cues (13). In addition, diabetes is often accompanied by changes in subjective mood states and emotional behavior, such as depression (14, 15). Previous studies have shown that exercise can improve cognitive impairment (16), appetite (17), and depression (18), while most of them have evaluated the brain function using questionnaires and scales. At present, functional magnetic resonance imaging (fMRI) is the mainstay of neuroimaging in cognitive neuroscience, providing a method to investigate the possible causal relationship between brain activity and behavior (19, 20). A recent clinical trial performed in Hong Kong reported that gut microbiota and metabolites mediated exercise-induced metabolic benefits to prediabetic men (21). Exercise affects the gut microbiota composition and microbial metabolites (22), while gut microbiota and their metabolites are involved in regulating behavior and brain activity, including stress response, emotion, pain, feeding behavior, and cognition (23, 24). However, there is a paucity of research elucidating whether the potential physiological benefits of exercise for diabetes mellitus are related to the modulation of the microbiota-gut-brain axis.

Herein, we introduce a detailed design of effects of exercise intervention on type 2 diabetes patients with abdominal obesity and low thigh circumference (EXTEND) study. We will enroll sedentary diabetes patients with abdominal obesity and low thigh circumference and explore the effect of combined aerobic and resistance exercise. The primary purpose of this trial is to clarify the role of exercise on abdominal obesity and loss of muscle mass, which will be accurately quantified using magnetic resonance imaging (MRI). Furthermore, many studies conducted on type 2 diabetes demonstrate that exercise intervention improves anthropometric, metabolic, and clinical indicators, with a lack of assessment of energy metabolism, diabetes complications, and brain health. There is an urgent need for more clinical researches with comprehensive evaluation and accurate measurement to investigate the role of exercise in type 2 diabetes. Thus, we aim to provide a multidimensional evaluation of the role of exercise through a broad assessment of clinical parameters, including anthropometric measurements, information of clinical status and lifestyle, biochemical indicators, psychosocial characteristics, accurate imaging examination, exercise-related indicators, and so on. Moreover, multi-omics analysis, such as metagenomics, metabolomics, proteomics, and single-cell sequencing, will be conducted. We believe that a comprehensive collection of clinical indicators and multi-omics data will provide a foundation for clinical research on diabetes, and the role of the microbiota-gut-brain axis in the benefits of exercise for diabetes will be elucidated.

## Methods and Analysis

### Study Design

**Figure 1:**
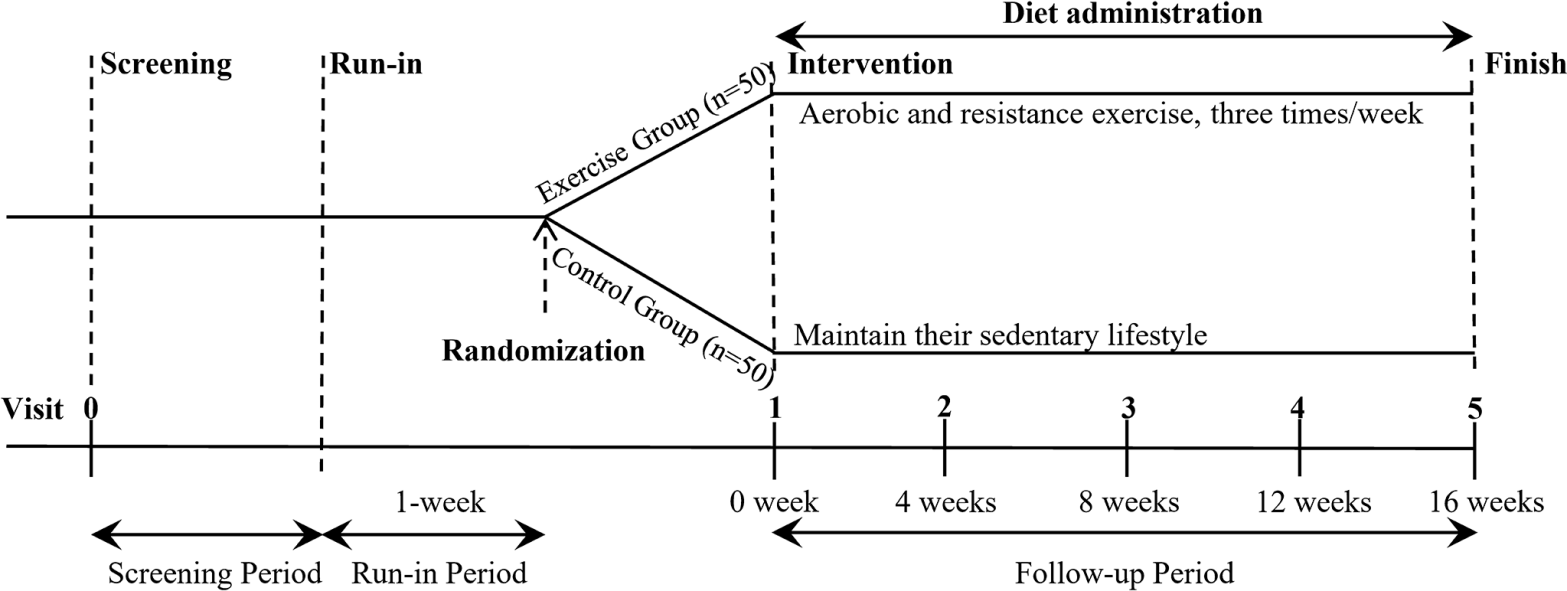
Overview of the study design. After screening and a 1-week run-in period, eligible participants are randomly assigned to either exercise group or control group (n = 50 per group) for 16-week intervention. During the whole trial, participants in exercise group receive combined aerobic and resistance exercise training three times a week, and control group maintain their sedentary lifestyle unchanged. All participants receive a diet administration during the intervention. In addition, participants take part in a follow-up visit every four weeks.

This study is a randomized controlled clinical trial. The participants will be randomized into two groups in a 1:1 ratio: exercise intervention and sedentary control group. The participants (50 participants per group) will undergo a 16-week intervention. Participants in the control group will maintain their sedentary lifestyle unchanged, and the exercise intervention group will receive combined aerobic and resistance exercise training. Moreover, all participants will be required to undertake a balanced diet to reduce the confounding effects of different dietary habits. The study design is outlined in **Figure 1**.

### Participants and Recruitment

The study will include adults aged 35-65 years of any sex, who have been diagnosed with type 2 diabetes mellitus and are not insulin-dependent. Potential participants will be recruited at the Shanghai Jiao Tong University Affiliated Sixth People’s Hospital. The inclusion and exclusion criteria are presented in **Table 1**. The potential benefits and risks of the study will be informed to the participants before screening. Written informed consent will be obtained from all participants. Physicians will conduct a preliminary clinical evaluation to screen eligible participants, including anthropometric measurement (e.g., height, weight, body mass index, and waist, hip, and thigh circumference), blood pressure, glycated hemoglobin (HbA1c), kidney function assessment, basic information (e.g., age, sex, menopause, education, and occupation), and the questionnaire information collection (lifestyle habits, family history, and disease and medication records). If participants meet all the criteria, they will be invited to enter a run-in period, which will be followed by randomization of participants who complete the run-in period.

**Table 1:** Inclusion and exclusion criteria.

Inclusion Criteria
Diagnosed with type 2 diabetes mellitus; Not more than three antidiabetic agents; The use of antidiabetic agents is stable for at least 6 weeks, and remains unchanged during the study intervention; Aged 35 to 65 years old; Abdominal obesity: waist circumference ≥ 90 cm for males and ≥ 80 cm for females; Body mass index ≤ 35 kg/m^2^; Waist to thigh ratio (WTR): male ≥ 1.7, female ≥ 1.6; Sedentary lifestyle (≤ 60 minutes of moderate-intensity activities per week).
**Exclusion Criteria**
Glycated hemoglobin < 6.5% or ≥ 9%; Insulin usage; Presence of one or more of the following complications: severe proliferative diabetic retinopathy or worse, macroalbuminuria (urine albumin-creatinine ratio ≥ 300 mg/g), or renal dysfunction (eGFR ≤ 60 ml/min/1.73m^2^); History of cardiovascular events, including myocardial infarction, percutaneous coronary intervention, unstable angina, heart failure, and cardiac insufficiency; History of cerebrovascular diseases, including hemorrhagic and ischemic stroke; Muscle, bone, or neuromuscular injuries, and so on that will prevent exercise training; Severe osteoporosis, or suboptimal bone density test results even after treatment; Pregnancy; Not able or unwilling to undergo magnetic resonance imaging, including claustrophobia, implantable cardioverter defibrillator and pacemaker;

10. Diagnosed depression or any mental illness rendering the patients unable to understand the nature, scope and schedule of the study; 11. atients who take drugs that are unstable or affect glucose metabolism.

### Randomization and Blinding

The randomization digital table is generated using a computer. Participants will be randomly assigned to the exercise or control group in a 1:1 ratio stratified by age and sex. The group assignment will be performed by an independent statistician who would be blinded to the recruitment, intervention, and evaluation of participants. Blinding of participants and researchers (who provide exercise instructions) to group assignment would not be possible due to the specific nature of the study. However, the researchers and statisticians who perform the follow-up assessments and statistical analyses will be blinded to group assignment.

### Data Collection and Follow-Up

Participants will participate in baseline, 4, 8, 12, and 16-week follow-up visits at the Department of Endocrinology and Metabolism, Shanghai Jiao Tong University Affiliated Sixth People’s Hospital. **Table 2** shows the schedule of the study process. The clinical assessments, measurements, and data collection will be conducted by independent researchers who have been trained before the study. Apart from the anthropometry and hematology examination, physicians will also perform a series of clinical assessments, including bioelectrical impedance analysis, MRI of abdomen and lower limbs, magnetic resonance spectroscopy, liver transient elastography, endothelial function ultrasound, flash glucose monitoring, heart rate variability analysis, and exercise-related tests (**Table 2**). The international physical activity questionnaire will be completed to evaluate habitual physical activity over the previous seven days, and a three-dimensional accelerometer tester (wGT3x-BT, Manufacturing Technology Inc, MTI, Florida, America) will be used to continuously monitor the basal physical activity over the following seven days on visit 1, 3, and 5. The dietary habits will be assessed through the three-day food record and food frequency questionnaire. In addition to assessment through conventional questionnaires (e.g., 100-mm visual analog scale, Patient Health Questionnaire-9, Generalized anxiety Disorder scale-7, Problem Areas in Diabetes scale, and Montreal Cognitive Assessment Scale), the brain health of the participants will be accurately evaluated by fMRI. All questionnaires will be filled by participants with the help of trained researchers during a face-to-face interview. Furthermore, blood, urine, stool, and saliva samples will be collected before and after intervention for further multi-omics analysis. Detailed methods of measurements and evaluations are described in the **Supplementary Material**.

**Table 2:** Schedule of the study process.

Time point	Screening	Visit 1	Visit 2	Visit 3	Visit 4	Visit 5
	-2 weeks ~ 0 week	0week	4 weeks	8 weeks	12 weeks	16 weeks
**Eligibility screening**	✓					
**Informed consent**	✓					
**Anthropometrics** (height, weight, body mass index, and waist, hip, and thigh circumference)	✓	✓	✓	✓	✓	✓
**Bioelectrical impedance analysis** (fat mass and percentage, lean mass and percentage)		✓	✓	✓	✓	✓
**Biochemical indicators** (liver enzyme, renal function, blood glucose, insulin, C peptide, and blood lipid)		✓		✓		✓
**HbA1c**	✓			✓		✓
**Cytokines** (FGF21, FGF19, FABP4, LCN2, adiponectin, etc.)		✓		✓		✓
**Appetite related hormones** (GLP-1, ghrelin, PYY, etc.)		✓		✓		✓
**Magnetic resonance imaging**		✓		✓		✓
**Functional magnetic resonance imaging**		✓				✓
**Magnetic resonance spectroscopy**		✓		✓		✓
**Liver transient elastography**		✓		✓		✓
**Endothelial function ultrasound**		✓		✓		✓
**Exercise-related tests** (cardiopulmonary fitness test, three-dimensional accelerometer test, muscle oxygen monitoring, physical fitness test)		✓		✓		✓
**Isolation of peripheral blood monocytes**		✓		✓		✓
**Heart rate variability analysis**		✓		✓		✓
**Flash glucose monitoring**		✓				✓
**Metagenomic analysis**		✓				✓
**Metabolomic analysis**		✓				✓
**Questionnaires** (International physical activity questionnaire, three-day food record, food frequency questionnaire, 100-mm VAS, PHQ-9, GAD-7, PAID, MoCA)		✓		✓		✓

FABP4, fatty acid-binding protein 4; FGF, fibroblast growth factor; GAD-7, Generalized anxiety Disorder scale-7; GLP-1, glucagon-like peptide-1; HbA1c, glycated hemoglobin; LCN2, lipocalin 2; MoCA, Montreal Cognitive Assessment scale; PAID, Problem Areas in Diabetes Scale; PHO-9, Patient Health Questionnaire-9; PYY, peptide YY; VAS, visual analog scale.

### Intervention

#### Exercise Intervention

Based on cardiopulmonary fitness and one repetition maximum, an individualized exercise training (combined aerobic and resistance exercises, three sessions per week) regimen will be developed. Each practice session will take 80 minutes, including 10 minutes of warm-up, 15 minutes of aerobic exercise, 10 minutes of resistance training, 10 minutes of rest, 15 minutes of aerobic exercise, 10 minutes of resistance training, and 10 minutes of relaxation. Aerobic and resistance exercises will include training with magnetic exercise bikes (HG-HRUB150T) and chest press/rowing (KY-701), abdominal and back muscle training (KY-705), and leg kick and hook (KY-703) machines (Nanjing Kuanyue Health Technology Co., Ltd, Nanjing, China). All aerobic and resistance training data will be recorded by an intelligent detection software system (Nanjing Kuanyue Health Technology Co., Ltd, Nanjing, China). The exercise training will be progressive and variable. Aerobic exercise programs will be divided into four training cycles (four weeks per cycle) and resistance exercise programs will be divided into three cycles. The differences in the training cycles will primarily be reflected in terms of the intensity and load. Details of exercise intervention are shown in **Table 3**.

**Table 3:** Protocol of exercise intervention.

Phase	Week	Intensity	Load
Aerobic Exercise			
I	1 – 4 weeks	40% VO_2max_	Males: 35 W; Females: 25 W
II	5 – 8 weeks	50% VO_2max_	Males: 40 W; Females: 30 W
III	9 – 12 weeks	60% VO_2max_	Males: 45 W; Females: 35 W
IV	13 – 16 weeks	70% VO_2max_	Males: 50 W; Females: 40 W
**Resistance Exercise**			
I	1 – 6 weeks	50% 1RM	1-2 weeks: 8 times/muscle group * 2 group
			3-4 weeks: 10 times/muscle group * 2 group
			5-6 weeks: 12 times/muscle group * 2 group
II	7 – 12 weeks	60% 1RM	7-8 weeks: 8 times/muscle group * 2 group
			9-10 weeks: 10 times/muscle group * 2 group
			11-12 weeks: 12 times/muscle group * 2 group
III	13 – 16 weeks	65% 1RM	13-14 weeks: 8 times/muscle group * 2 group
			15-16 weeks: 10 times/muscle group * 2 group

Exercise intensity will be monitored and instructed using Rating of Perceived Exertion, with scope definition of 12 - 16. The intensity of exercise can be adjusted according to the weight and actual condition of participants. 1RM, 1 repetition maximum; VO_2max_, maximum oxygen uptake.

Participants in the exercise group will take part in exercise training at the Shanghai Jiao Tong University Affiliated Sixth People’s Hospital under the guidance of professional coaches. The duration and intensity of each exercise session will be recorded, and the coach will make personalized adjustments to the exercise intensity of each participant. The blood pressure, heart rate, and finger-stick blood glucose of participants will be recorded before and after the exercise session.

#### Diet Intervention

Diet intervention will be administered during the study period. The purpose of diet administration is to control the dietary intake of each participant and reduce the confounding effect of different dietary habits. The ideal body weight (kg) of an individual is equal to the height (cm) minus 105, and the recommended daily energy intake (kcal) is equal to the ideal body weight multiplied by 25 (kcal/kg). During the study, all participants will prepare and consume food based on a recommended diet chart, which will be prepared by a nutritionist according to the Dietary Guidelines for Chinese Residents (25) and Dietary Guidelines for Type 2 diabetes in China (26). To increase the dietary compliance, participants will be asked to take photos of each meal and record the type and volume of food that they consume on the Boohee Nutrition Mini Program (Shanghai Mint Health Technology Co., Ltd, Shanghai, China). Based on these records, the Boohee Nutrition Mini Program will automatically calculate and analyze the total energy intake, as well as protein, carbohydrate, fat, and other nutrient intake. Investigators will assess the dietary record on a weekly basis to assess adherence to the dietary regimens, and upload the dietary record to the data repository for further analysis.

### Study Outcomes

#### Primary Outcomes

Change in body fat mass measured by bioelectrical impedance analysis (DBA-210, software version 3.5, Donghuayuan Medical, Jilin, China).

#### Secondary Outcomes

Secondary outcomes include change rate of body fat mass, and changes in lean mass and percentage, anthropometric indicators (body weight, body mass index, and waist, hip, and thigh circumference), clinical biochemical indicators (HbA1c, insulin sensitivity, liver enzyme, renal function, blood lipid, blood glucose, serum insulin and C peptide), brain health (appetite, mood, and cognitive function), immunologic function, metagenomics, metabolomics, energy expenditure, cardiopulmonary fitness (maximum oxygen uptake, maximum oxygen uptake/predicted oxygen uptake), exercise-related indicators (physical activity, grip, flexibility, lower limb muscle endurance and strength, static and dynamic balance function, and muscle oxygen), fatty liver, cytokines (fibroblast growth factor [FGF] 21, FGF19, adiponectin, fatty acid-binding protein 4, and lipocalin 2), vascular endothelial function, autonomic nervous function, and glucose fluctuation.

### Data Management and Monitoring

The paper case report form for each participant will be filled by trained researchers. It will be used to collect information and will be kept strictly confidential for research purposes. Researchers will keep files intact, stored in a fixed place and locked at the Department of Endocrinology and Metabolism, Shanghai Jiao Tong University Affiliated Sixth People’s Hospital. All data will be scrutinized and double-entered by researchers. After the database is established and confirmed *via* blind audit, it will be locked and will not be changeable.

This study will be monitored by an independent Data Monitoring Committee comprising of specialists who are independent of this trial. Serious adverse events will be reported immediately to Data Monitoring Committee, principal investigator, and the Ethics Committee of the Shanghai Sixth People’s Hospital. The Data Monitoring Committee will audit the trial processes and data, and be responsible for discontinuing the trial.

### Sample Size Estimation

We will conduct an exercise intervention in two groups. The reduction in body fat mass will be the primary outcome. According to previous studies (27), the calculated sample size is 40 for each group to detect a 1.2 kg difference in body fat mass between two groups, with a significance level of 0.05 (α), statistic power of 80% (1-β), and standard deviation of 1.9 kg. Assuming a 20% loss to follow-up, a sample size of 50 will be required per group. Therefore, a total of 100 patients will be recruited.

### Statistical Analysis

Both the intention-to-treat and full analysis set analyses will be performed to examine effectiveness of exercise intervention on type 2 diabetes patients with abdominal obesity and low thigh circumference. Statistical analyses will be performed using SPSS 25.0 software (SPSS, Inc, Chicago, IL). Multiple imputations will be used to process missing values in the data. Data will be expressed as mean ± standard deviation, median (interquartile range) and percentage appropriately. Kolmogorov-Smirnov test will be used to determine the normal distribution of data. All statistical tests will be two-tailed, with a 0.05 significance level.

Baseline characteristics will be compared between groups by Student’s unpaired t-test for normal distributed data, Mann-Whitney test for non-normal distributed data, and Chi-squared test for categorical data, respectively. Comparisons between baseline and different time points (4, 8, 12, and 16-week follow-ups) in each group will be performed using paired t-test for normal distributed data and Wilcoxon signed-rank test for non-normal distributed data. Changes in clinical and multi-omics data before and after the intervention will be compared between groups using analysis of covariance, with baseline variable levels as covariates. Mixed-effect models will be performed to evaluate the effect of exercise intervention on clinical indicators. Additionally, multiple linear regression will be performed for sensitivity analysis of the main clinical variables, adjusting for age, sex, duration of diabetes, and diet. The composition of the gut microbiota will be analyzed from the metagenomic sequencing data using MetaPhlAn calculating software. Differences in the abundance of microbiota species before and after the intervention will be compared using Wilcoxon test. Differences in the abundance of microbiota between groups will be compared using Mann-Whitney test. Correlation between abundance of microbiota, metabolite levels, and clinical indicators will be analyzed using Spearman correlation and Partial correlation analysis.

### Patient and Public Involvement

During the development of the study protocol, patients and public were not directly interviewed. Patients were not involved in the study design, management, and conduct. The results of this study will be informed to participants by their physicians.

## Discussion

### Multidimensional Evaluations of Exercise for Diabetes

Type 2 diabetes is a chronic metabolic disease characterized by hyperglycemia, insulin resistance, β-cell function declines, and abnormal fat and protein metabolism (28). It is worth noting that diabetes is commonly accompanied with abnormal energy metabolism and dysbiosis of gut microbiota (29), and also coexists with many other diseases (30), including micro- and macro-vascular diseases, depression, cognitive impairment, and autonomic neuropathy. Over the past decade, the benefits of exercise in patients with type 2 diabetes have been well demonstrated (31–33). Clinical studies on exercise intervention in type 2 diabetes have mostly investigated the effect of exercise on glucose and lipid metabolism, obesity-related indicators, physical activity, and quality of life (34, 35). However, considering the complex pathophysiology of diabetes, the effect of exercise on diabetes complications, energy metabolism, and gut microbiota needs further investigation. Therefore, clinical trials with multidimensional evaluations and accurate measurement methods are needed to completely assess the action of exercise on diabetes.

In this study, we will attempt to comprehensively evaluate the physiological benefits of exercise from the following aspects. First, anthropometrics and body composition will be accurately measured. Second, biochemical variables (including HbA1c, insulin sensitivity, liver enzyme, renal function, blood lipid, and blood glucose), physical fitness, cardiopulmonary fitness, and energy expenditure will be measured to assess the metabolic effects of exercise. Third, vascular endothelial function, immunologic function, and diabetes complications (including diabetic nephropathy and autonomic neuropathy) will be evaluated. Fourth, flash glucose monitoring system will be used to evaluate the glucose fluctuation of diabetes patients. Fifth, conventional mental health-related questionnaires (including 100-mm visual analog scale, Patient Health Questionnaire-9, Generalized anxiety Disorder scale-7, Problem Areas in Diabetes scale, and Montreal Cognitive Assessment Scale) and fMRI will be used to assess the appetite, emotion, and cognition of diabetes patients. Lastly and importantly, the blood, urine, stool, and saliva samples will be collected for further multi-omics analysis.

### Type 2 Diabetes Patients With Abdominal Obesity and Low Thigh Circumference

Abdominal obesity is an important risk factor of the development of type 2 diabetes (36). A low thigh circumference is also associated with an increased risk of insulin resistance and diabetes (6). Low femoral subcutaneous fat is associated with unfavorable glucose levels (5). Many diseases, such as diabetes mellitus, cancer, chronic obstructive pulmonary disease, or heart failure, can cause muscle wasting, which is considered as secondary sarcopenia (8). Sarcopenia is a geriatric disease characterized by a progressive loss of skeletal muscle mass and loss of muscle function (37), and is associated with disability, osteoporosis, and an increased risk of cardiometabolic diseases (38). Diabetes is common among the elderly population, affecting approximately 25% of people over 65 years of age (39). Thus, further studies are needed on sarcopenia in individuals with diabetes. This study will enroll type 2 diabetes patients with abdominal obesity and low thigh circumference. We aim to clarify the role of combined aerobic and resistance exercise on abdominal obesity and muscle mass. Previous clinical studies have reported that exercise can improve the body composition of diabetes patients (40–43), while few of them have assessed it using accurate measurement methods, such as MRI. In this study, apart from the measurement of anthropometric indicators, fat mass and percentage, and lean mass and percentage, we will also accurately quantify the area of abdominal and femoral adipose tissue and femoral muscle through MRI. We hypothesize that exercise training can improve the body composition of diabetes patients, with reduced visceral fat accumulation and increased muscle mass.

### Exploration of the Microbiota-Gut-Brain Axis

Type 2 diabetes mellitus is associated with cognitive decline in all age groups, which affects cognitive domains including memory, information processing speed, attention, and executive functioning (44). Researches have reported that age, education, and co-existence with mild cognitive impairment, depression, and vascular diseases are associated with an increased risk of dementia in diabetes patients (45–47). Exercise can induce benefits to type 2 diabetes related cognitive impairment and dementia (16). Findings from a large, long-term, randomized controlled trial have suggested that a multidomain intervention (diet, exercise, cognitive training, and vascular risk monitoring) can improve or maintain cognitive function in at-risk elderly people (48). Additionally, type 2 diabetes is related with depression and alterations in appetite-related processes (14, 49), both of which can be influenced by exercise training (17, 18, 49). The role of microbiota in regulating gut-brain function contributes to the appreciation of the microbiota-gut-brain axis (23). The gut microbiota produce and release various molecules and metabolites, which in turn mediate the diverse actions of the gut microbiota, including the regulation of energy metabolism, immune system (50), appetite sensation (51), and brain function (52, 53). Evidence from both animal and clinical studies demonstrates that exercise improves intestinal hemostasis by influencing the diversity and composition of the microbiota and metabolite levels (54, 55). However, whether the physiological benefit of exercise in individuals with diabetes is related to the modulation of the microbiota-gut-brain axis is yet to be fully explored.

In this study, we will investigate the role of the microbiota-gut-brain axis in the metabolic benefits of exercise in patients with diabetes. Apart from the related questionnaires and scales, the appetite and cognitive function of participants will be accurately assessed through resting state and task fMRI scans, which include food cue visualization- (non-food, high-calorie, and low-calorie food pictures), inspection time-, n-back-, and flanker tasks. Meanwhile, appetite hormones (including glucagon-like peptide-1, ghrelin, and peptide YY) and cytokines (including FGF21, FGF19, fatty acid-binding protein 4, lipocalin 2, and adiponectin) levels will be measured. Furthermore, metabolomics and metagenomics analysis will be performed to provide support for the actions of gut microbiota and microbial metabolites. The relationship between clinical symptoms, brain health, and multi-omics will be further analyzed to investigate the modulation of the microbiota-gut-brain axis in the benefits of exercise for diabetes.

In conclusion, this study is the first randomized clinical trial to investigate the effect of combined exercise on diabetes patients with abdominal obesity and low thigh circumference. The findings will provide a comprehensive evaluation of exercise in patients with diabetes, reveal the underlying role of the microbiota-gut-brain axis in the physiological benefits of exercise for diabetes, and provide a new approach to research the modulation of the microbiota-gut-brain axis.

## Ethics Statement

The studies involving human participants were reviewed and approved by the Ethics Committee of the Shanghai Sixth People’s Hospital (2019-099). The patients/participants provided their written informed consent to participate in this study.

## Author Contributions

WJ and HL conceived the study. DL, YZ, LW, YNZ, BS, WJ and HL initiated the study design and contributed to protocol development and implementation. JG, XY, HY, RH, TM, YCZ, QG, QF, and YZ helped in sample size calculation, statistical analysis plan, and data collection and critically reviewed the manuscript. All authors contributed to the article and approved the submitted version.

## Funding

This work is supported by the Excellent Young Scholars of National Natural Science Foundation of China (NSFC) (82022012), General Program of NSFC (81870598), Two Hundred Program from Shanghai Jiao Tong University School of Medicine (20191830), and Innovative research team of high-level local universities in Shanghai (SHSMU-ZDCX20212700) to HL; Shanghai Municipal Key Clinical Specialty (2017ZZ01013), Shanghai Key Clinical Specialty Construction Project (LY01.05.02), and the National Key Research and Development Program of China (2018YFA0800402) to WJ; the National Natural Science Foundation of China (82100879), Shanghai Pujiang Program (2020PJD044), and the Exploration Fund Grant of Shanghai Sixth People’s Hospital (ynts202003) to LW.

## Conflict of Interest

The authors declare that the research was conducted in the absence of any commercial or financial relationships that could be construed as a potential conflict of interest.

## Publisher’s Note

All claims expressed in this article are solely those of the authors and do not necessarily represent those of their affiliated organizations, or those of the publisher, the editors and the reviewers. Any product that may be evaluated in this article, or claim that may be made by its manufacturer, is not guaranteed or endorsed by the publisher.
