# Supplementary material for: Effects of Exercise Intervention on Type 2 Diabetes Patients With Abdominal Obesity and Low Thigh Circumference (EXTEND): Study Protocol for a Randomized Controlled Trial
Source: Front Endocrinol (Lausanne). 2022 Jul 12;13:937264. doi: 10.3389/fendo.2022.937264 (PMC9317299; doi:10.3389/fendo.2022.937264)
Supplement: Supplementary file 1 [file DataSheet_1.pdf]

Supplementary Material

1 Measurements and Evaluations

1.1 Anthropometrics and Vital Sign

After an overnight fasting for at least 10 hours, participants will receive general information collection at the Department of Endocrinology and Metabolism, Shanghai Jiao Tong University Affiliated Sixth People's Hospital. Height, weight, waist circumference, hip circumference and thigh circumference will be measured. Vital signs, including blood pressure and heart rate, will also be recorded.

1.2 Body Composition

Body composition will be measured using bioelectrical impedance analysis (DBA-210, software version 3.5, Donghuayuan Medical, Province Jilin, China), with all external metal items removed from the participants. The whole-body fat mass, fat mass percentage, lean mass and lean mass percentage will be recorded.

1.3 Magnetic Resonance Imaging

The 3.0T magnetic resonance imaging (MRI) (Ingenia, Philips Medical System, The Netherlands) will be used to scan the area of abdominal visceral and subcutaneous adipose tissue, femoral subcutaneous adipose tissue, and the muscle area of hip, thigh and lower leg. MRI scans will be performed by an experienced radiologist using standard array coils at the parallel part of the abdomen between the L4 and L5 vertebrae in the supine position, the section of the femoral head, the midpoint of the line between the anterior superior iliac spine and the superior edge of the patella, and the thickest level of the lower leg. Images will be segmented and calculated for visceral fat area, abdominal subcutaneous fat area, femoral subcutaneous fat area, and muscle area of hip, thigh and lower leg using SliceOmatic image analysis software (version 4.2; Tomovision Inc., Canada).

1.4 Magnetic Resonance Spectroscopy

Intrahepatic triglyceride content will be quantified by magnetic resonance spectroscopy using a 3.0T MRI (Ingenia, Philips Medical System, The Netherlands). The right branch of the portal vein will be selected to enter the liver level, and the fat fractions in the right anterior, right posterior and left lobe regions will be measured. The region of interest (ROI) cross-sectional area is 290-310 mm^2, and the intrahepatic vessels will be avoided as much as possible during the measurement. All scans and measurements will be performed by the same operator, taking the average of the three ROI measurements (left lobe, right anterior lobe, right posterior lobe) as the final measurement levels.

1.5 Functional Magnetic Resonance Imaging

Functional magnetic resonance imaging (fMRI) will be conducted using 3.0T MRI scanner (Ingenia, Philips Medical System, The Netherlands) and brain functional audiovisual stimulation system (SA-9939, Shenzhen Sinorad Medical Electronics Co., Ltd, Guangdong, China). In cooperation with psychiatrists, we will use fMRI to evaluate the changes of appetite and cognitive function after intervention (1, 2).

Before the experiment, the participants should be fasting for at least 6 hours in advance. The trained researcher will conduct pre-experiment practice with participants before the official test. The experimental procedure will be introduced to the participant, including the duration, procedure and matters needing attention. Participants will be randomly assigned the task number, and the screen and buttons in the MRI room will be checked to ensure normal operation. In the nuclear magnetic chamber, the participants will take the supine position, with the head fixed with vacuum pillow and belt to reduce the head motion. The brain function will be assessed using resting-state and task fMRI. The appetite task is to evaluate the participants' response to pictures stimuli (non-food, low-calorie food and high-calorie food pictures) in a fasting state and half an hour after a standard meal (3). The cognitive task consists of three parts: the inspection time task to evaluate response speed, the n-back task to evaluate working memory, and the flanker task to evaluate cognitive control and conflict inhibition. Finger-stick blood glucose and 100-mm visual analog scale (VAS) for appetite sensations will be measured before and after the fMRI scan.

1.6 Cardiopulmonary Fitness Test and Energy Expenditure Evaluation

To determine the intensity of exercise training and evaluate the effects of exercise intervention on cardiopulmonary fitness, we will work with the cardiologist to perform a cardiopulmonary fitness test. In brief, the exercise test will be conducted with a bicycle ergometer until exhaustion. According to the health status of each participant, the work load will increase continuously and linearly at a rate of 10 − 25 W/min. Respiratory gas exchange will be recorded continuously during the exercise test using Sensor Medics Vmax29 Cardiopulmonary Exercise Testing System (Sensor Medics, Yorba Linda, California, USA) (4, 5). VO_{2max}, anaerobic threshold VO_2, and energy expenditure will be calculated. During the test, subjective symptoms, blood pressure, electrocardiogram and heart rate will also be observed and recorded. In addition, the Sensor Medics Vmax29 Cardiopulmonary Exercise Testing System (Sensor Medics, Yorba Linda, California, USA) will be used to measure the subjects' pulmonary function, including forced vital capacity, forced expiratory volume in one second and maximal voluntary ventilation, etc.

1.7 Liver Transient Elastography

Transient elastography will be performed to evaluate severity of hepatic steatosis and fibrosis using the FibroScan® device (EchoSens, Paris, France) (6). The patients will lie on their backs, with right arm in maximum abduction to facilitate access to the right liver lobe by intercostal approach. Before transient elastography, two-dimensional ultrasound assisted point location will be conducted to locate a liver portion free of large vessels, substantive placeholders, gallbladder, superior border of right kidney and others. The examination will be performed using an M probe or XL probe according to the EASL-ALEH Clinical Practice Guidelines (6). Liver stiffness measurements and controlled attenuation parameter will be measured by an experienced ultrasound physician who is not aware of the group assignment. For each patient, the ultrasound physician will perform the test with ten successful shots and then record the median values.

1.8 Endothelial Function Ultrasound Examination

High resolution ultrasound equipment (UNEXEF38G, UNEX Corporation, Japan) and high frequency transducer will be used to obtain longitudinal images of the brachial artery. The test will be performed by experienced ultrasound physician. Measurements will be made in a dark and quiet room with temperature of 20-25℃. After participants rest in supine position for 10 minutes, the cuff for expelling

blood, the cuff of the sphygmomanometer and the ECG sensors are installed on the patient's arms. Use the robotic arm to locate the H-type probe to the clearest part of the media of the long axis, and measure the internal diameter of the basic brachial artery and blood flow in the quiet state. After that, inflate the cuff until it is at least 50 mmHg above the systolic blood pressure for 5 minutes to produce ischemic stimulation. Then the cuff will be loosened and the images of brachial artery will be recorded continuously from 30s before cuff releasing to 2 minutes after cuff releasing (7). The baseline diameter, absolute change and percent change in brachial artery diameter will be taken as the evaluation results of endothelial function. The flow-mediated dilation is defined as the percentage of increase in peak diameter to baseline diameter. The position of the sensor and the arm remains the same throughout the measurement.

1.9 Three-dimensional Accelerometer Test

Three-dimensional accelerometer tester (wGT3x-BT, Manufacturing Technology Inc, MTI, Florida, America) will be used to evaluate the levels of physical activity. GT3x is a three-axis accelerometer with small motion sensor, which reflects the levels of physical activity by measuring acceleration on vertical axis, horizontal axis and sagittal axis. Participants will be required to wear accelerometer on the abdomen for 7 days (valid wearing time consist of three working days and one weekend), except sleeping, bathing or other water activities. Daily physical activity will be quantified as minutes and percentages of sitting, low-, moderate- and high-intensity physical activity using the ActiLife Data Analysis Platform (version 6.13, Manufacturing Technology Inc, Florida, America).

1.10 Muscle Oxygenation

We will evaluate changes in muscle oxygenation during cardiopulmonary fitness test through a real-time wireless muscle blood oxygen monitoring system (PortaMon, Artinis Medical Systems, BV, The Netherlands). The muscle blood oxygen monitoring system is a wireless portable device for non-invasive measurement of muscle blood oxygen based on near-infrared spectroscopy. The main indexes include muscle oxygen saturation, blood oxyhemoglobin, deoxyhemoglobin, total hemoglobin and oxygen saturation index.

1.11 Physical Fitness Test

Physical fitness test will be performed by the exercise coaches and rehabilitation physicians, including grip test, flexibility test, lower limb muscle endurance test, knee joint flexors and extensors strength test, static and dynamic balance function test.

For grip test, participants will be encouraged to exert their maximal effort to grip the dynamograph twice using dominant hand, and the maximum values will be recorded. The flexibility will be measured twice through sit and reach, and the maximum value will be recorded to the nearest 0.1 cm. The muscle endurance of lower limb will be tested through stand-to-sit test (8). The number of completed actions in 30 seconds will be recorded.

Knee joint flexors and extensors strength test will be performed using isokinetic strength test system (Multi-joint isokinetic strength test and training system A8-2, Guangzhou Yikang medical equipment industry Co., Ltd, Guangzhou, China). Using centripetal-centripetal mode, we will select 60 (°)/s for maximum strength test and 180 (°)/s for quick strength test. Participants will perform quadriceps and hamstrings tests on the knee at 90° flexion to full extension of the knee. Peak torque (N•m), peak torque/body weight (N•m/kg), average power (J) and total work (J) will be recorded and analyzed using specialized software.

The static balance function will be tested using a balancing apparatus (Balance-B, Shanghai NCC Electronic Co., Ltd, Shanghai, China). The length and speed of center-of-mass displacement in the anterior-posterior and right-left directions will be recorded. The stand-to-walk test will be used to evaluate the dynamic balance function of the participants. When the coaches give the "start" command, the subjects immediately stand up and walk forward as fast as possible, turn over at 3 meters away from the seat, then return to the chair again. The time from the back leaving the chair to the back touching the chair will be recorded.

1.12 Heart Rate Variability

Heart rate variability analysis is a measurement of heart rate change, which reflects the degree of fluctuation of successive heartbeat interval length. The autonomic nervous function will be evaluated by Medeia 3000 heart rate variability analysis system (Guangzhou Meideia Medical Technology co, Ltd, Guangzhou, China). The test will be carried out in the quiet environment with the least number of participants. Considering the possible influence of circadian rhythm, the analysis of heart rate variability of all participants will be carried out in the morning. The clinical characteristics of the individuals need to be evaluated before the analysis of heart rate variability (9). The device will capture the R–R intervals through the attachment of electrodes to the arms.

1.13 Flash Glucose Monitoring

The flash glucose monitoring (FGM) system (FreeStyle Libre H, Abbott Diabetes Care Ltd.) will be installed in all participants to monitor glucose levels of interstitial fluid for 14 consecutive days. The sensor will be inserted into the subcutaneous tissue of upper arm. Finger-stick blood glucose levels will be measured to calibrate the glucose value of the FGM.

1.14 Blood Samples Collection

Blood samples will be collected from participants in fasting state and postprandial time points after a standard meal (instant noodles: 1566 KJ including 68.4 g carbohydrate and 10.4 g protein) to measure blood glucose, insulin, C peptide and biochemical test. Blood samples will be drawn respectively at 0min, 30min, 60min, 90min, and 120 minutes before and after eating noodles. Centrifuge at 4000 rpm, 4℃ for 10 minutes to isolate serum. Biochemical tests will be conducted using the automatic analyzer (Hitachi 7600-020, Tokyo, Japan). The serum for cytokines will be stored at -80℃ until further measurement. The concentrations of serum cytokines (including fibroblast growth factor 21, fibroblast growth factor 19, adiponectin, fatty acid-binding protein 4, lipocalin 2, etc.) will be quantitatively determined by enzyme-linked immunosorbent assay.

1.15 Quantitative Determination of Appetite-related Hormones in Blood Samples

The blood for glucagon-like peptide-1 and ghrelin will be collected with EDTA tubes containing dipeptidyl peptidase IV inhibitor (DPP IV inhibitor, 20 µl; cat# DPP4-010, Millipore, USA) and protease inhibitor 4-(2-aminoethyl) benzenesulfonyl fluoride hydrochloride (AEBSF, 20 µl; 200 mg/mL; cat# 11429868001, Roche Applied Science, Almere, Netherlands), respectively. Centrifuge at 4000 rpm, 4℃ for 10 minutes to isolate plasma. The blood for peptide YY will be collected with plain blood collection tube, and then the tube will be centrifuged at 4000 rpm, 4℃ for 10 minutes to isolate serum. The blood for appetite-related hormones will be stored at -80℃ until further measurement.

1.16 Isolation of Human Peripheral Blood Mononuclear Cells

The blood will be collected using BD Vacutainer® CPT™ Cell Preparation Tube with Sodium Citrate (REF 362761). After centrifuge the tube at 1500 rcf and room temperature for 30 minutes, the mononuclear cells and platelets will be collected at the plasma/density solution interface. Wash the cells with PBS, and take 10 μl mixture to test the cell viability and count the living cells. Then, re-suspended the cells in the cryopreservation solution, and transfer the mixture into the frozen tube for liquid nitrogen long-term preservation.

1.17 Stool, Urine and Saliva Samples Collection

The stool samples will be collected using a commercial tube with DNA stabilizer (STRATEC Molecular, Berlin, Germany). The participants will be advised to sample approximately 2-3 grams of feces, and deliver the sample to the hospital within 30 minutes to 1 hour or freeze the sample in the fridge until they go to hospital. The stool samples will be stored at -80℃ until further analysis.

The participants will be required to collect 24 hours urine one day before the visit, record the total urine volume and transfer 10 ml urine to the centrifuge tube for the 24-hour urea nitrogen and protein quantity. The participants will also collect 10 ml morning urine on the day of the visit and put it in another centrifugal tube for further metabonomic analysis. The collected urine will be centrifuged at 3000 rcf, 4℃ for 10 minutes, and then the supernatant will be collected and stored at -80℃ until further analysis.

The saliva samples will be collected by chewing cotton swabs matched with saliva collection tube (Salivette, Germany). After chewing the cotton swab 50 to 60 times in 1 minute, vomit it to the collection tube, and then the tube will be centrifuged at 3000 rpm, 4℃ for 15 minutes. The supernatant of the saliva sample will be collected and stored at -80℃ until further analysis.

2 References

1. van Bloemendaal L, RG IJ, Ten Kulve JS, Barkhof F, Konrad RJ, Drent ML, et al. GLP-1 receptor activation modulates appetite- and reward-related brain areas in humans. *Diabetes* (2014) 63(12):4186-96. doi: 10.2337/db14-0849
2. Imburgio MJ, Banica I, Hill KE, Weinberg A, Foti D, MacNamara A. Establishing norms for error-related brain activity during the arrow Flanker task among young adults. *Neuroimage* (2020) 213:116694. doi: 10.1016/j.neuroimage.2020.116694
3. Ten Kulve JS, Veltman DJ, van Bloemendaal L, Barkhof F, Drent ML, Diamant M, et al. Liraglutide Reduces CNS Activation in Response to Visual Food Cues Only After Short-term Treatment in Patients With Type 2 Diabetes. *Diabetes Care* (2016) 39(2):214-21. doi: 10.2337/dc15-0772
4. Marra M, Sammarco R, De Filippo E, De Caprio C, Speranza E, Contaldo F, et al. Resting Energy Expenditure, Body Composition and Phase Angle in Anorectic, Ballet Dancers and Constitutionally Lean Males. *Nutrients* (2019) 11(3). doi: 10.3390/nu11030502
5. Carvalho MR, Sato EI, Tebexreni AS, Heidecher RT, Schenkman S, Neto TL. Effects of supervised cardiovascular training program on exercise tolerance, aerobic capacity, and quality of life in patients with systemic lupus erythematosus. *Arthritis and rheumatism* (2005) 53(6):838-44. doi: 10.1002/art.21605

6. European Association for Study of Liver, Asociacion Latinoamericana para el Estudio del Higado. EASL-ALEH Clinical Practice Guidelines: Non-invasive tests for evaluation of liver disease severity and prognosis. *Journal of hepatology* (2015) 63(1):237-64. doi: 10.1016/j.jhep.2015.04.006

7. Corretti MC, Anderson TJ, Benjamin EJ, Celermajer D, Charbonneau F, Creager MA, et al. Guidelines for the ultrasound assessment of endothelial-dependent flow-mediated vasodilation of the brachial artery: a report of the International Brachial Artery Reactivity Task Force. *J Am Coll Cardiol* (2002) 39(2):257-65. doi: 10.1016/s0735-1097(01)01746-6

8. Nakamura K, Nagasawa Y, Sawaki S, Yokokawa Y, Ohira M, Sato Y. An Incremental Sit-to-Stand Exercise for Evaluating Physical Capacity in Older Patients with Type 2 Diabetes. *The Tohoku journal of experimental medicine* (2019) 249(4):241-8. doi: 10.1620/tjem.249.241

9. Catai AM, Pastre CM, Godoy MF, Silva ED, Takahashi ACM, Vanderlei LCM. Heart rate variability: are you using it properly? Standardisation checklist of procedures. *Braz J Phys Ther* (2020) 24(2):91-102. doi: 10.1016/j.bjpt.2019.02.006
